# Breast self-examination practice and predictors among female secondary school teachers in Addis Ababa, Ethiopia: using the health belief model

**DOI:** 10.1186/s12905-022-01904-w

**Published:** 2022-07-29

**Authors:** Bisrat Tewelde, Mulugeta Tamire, Mirgissa Kaba

**Affiliations:** 1 Department of Environmental Health and Behavioral Sciences, School of Public Health, Mekelle University, Mekelle, Ethiopia; 2 Department of Preventive Medicine, School of Public Health, Addis Ababa University, Addis Ababa, Ethiopia

**Keywords:** Breast self-examination, Female teachers, Health belief model, Ethiopia

## Abstract

**Background:**

Breast cancer is the most frequently diagnosed reproductive organ cancer among women in Ethiopia. Even though breast self-examination (BSE) is shown to be the least expensive, less time-consuming, and non-invasive screening method, the practice of breast self-examination in Ethiopia is poor. Therefore this study aimed to assess breast self-examination practice and predictors among female secondary school teachers using the Health Belief Model.

**Materials and methods:**

An institution-based, cross-sectional study was conducted among 589 female secondary school teachers in Addis Ababa, Ethiopia. A self-administered questionnaire containing socio-demographic characteristics, sources of information, knowledge, perception on breast self-examination, and BSE practice was prepared based on the Champion's revised Health Belief Model and used as a data collection instrument. Multi-variable binary logistic regression was employed to identify the predictors of breast self-examination practice with significance set at *p* < 0.05 by controlling possible confounders.

**Result:**

Breast self-examination was practiced by 43.6% of female secondary school teachers. Television and radio were the commonest sources of information about breast cancer and breast self-examination. Personal history of breast problem (AOR 3.27, 95% CI 1.13–9.45), teaching experience (AOR 2.46, 95% CI 1.33–4.56), knowledge (AOR 1.06, 95% CI 1.01–1.12) and perceived self-efficacy (AOR 1.07, 95% CI 1.01–1.12) were significantly associated with BSE practice.

**Conclusion:**

The practice of breast self-examination was found to be low. Perceived self-efficacy, personal history of breast problems, and the knowledge level of female teachers were factors associated with the practice of BSE. This suggests the need for educational programs to enhance knowledge regarding breast cancer and improve the practice of breast self-examination.

## Background

Breast cancer is the most frequently diagnosed cancer in women and in developing countries is the leading cause of cancer death among women and the second-leading cause of cancer death (following lung cancer) among women in developed countries [1, 2]. Worldwide, it is the most commonly diagnosed cancer among women with nearly 1.7 million new cases diagnosed annually accounting for 25% of all new cancer cases in women. It is the top common female cancer in Sub Saharan Africa accounting for 25.5% [2].

In Ethiopia, breast cancer is the most frequently diagnosed reproductive organ cancer (ROCs) and according to Addis Ababa city cancer registry, from 5701 registered cancer cases from 2011 to 2014, is the most commonly leading cancer among females accounting for 33% of the cases followed by cervix uteri (17%) [3].

Advanced breast cancer is more difficult to manage often with poor prognosis [4]. Therefore, early diagnosis remains an important strategy in improving breast cancer outcomes and survival particularly in low-and middle-income countries (LMICs) where women present to a health care facility where the disease is diagnosed in the late stages and resources required for treatment are limited. Evidence shows that breast cancer deaths can be reduced significantly if the tumor is discovered at an early stage [5, 6].

Early detection through screening in asymptomatic women aims to reduce mortality as well as the morbidity associated with advanced stages of the disease. The American Cancer Society (ACS) recommends early detection methods which vary depending on the woman's age including mammography and clinical breast examination (CBE) and breast self-examination (BSE). The ACS recommends all women should become familiar with both the appearance and feel of their breasts and report any changes promptly to their physician [1, 7].

BSE is when a woman systematically palpates each breast using her contralateral hand, with her ipsilateral arm raised above her head performing the examination in both lying and standing or sitting positions [7]. The goal of regular BSE is to detect palpable tumors and increase awareness of normal breast composition, so there is increased awareness of changes that may be detected. Therefore, women beginning in their 20s should be told about the benefits and limitations of BSE [8].

It has been shown that approximately 71% of breast cancer cases in women younger than 50 years of age are found by women themselves [9]**.** Even though BSE is shown to be the least expensive, less time consuming and non-invasive screening method, various studies conducted in Ethiopia showed that the practice of breast self-examination is poor [10–13] and the most commonly mentioned reasons for not practicing this behavior were lack of knowledge of the technique of performing BSE, not having breast-related problem or symptoms, afraid of being diagnosed with breast cancer and no advice or recommendation from HCWs [10, 11, 13–15].

The Health Belief Model (HBM) is a psychosocial model that assumes the perception of individuals is the best predictor of their behavior [16]. However, it has not received enough attention particularly among female teachers considered as a target group for information about breast cancer and other health issues as this group of educated women are seen as role models both on the school campus and in the community [17, 18]. However, in our study area there is a lack of evidence regarding the perception of female secondary school teachers regarding breast cancer and BSE in our study area. This study will therefore address modifiable factors for the poor practice and female teachers’ perception affecting the practice of BSE based on the HBM framework. The findings of this study may assist health-care providers, health educators and other concerned bodies in addressing the factors that determine women’s participation in BSE through effective strategies and programs. Therefore, this study aims to assess practice BSE practice and predictors among female secondary school teachers applying the Health Belief Model.

## Materials and methods

### Study design and area

Institution-based cross-sectional study was conducted in Addis Ababa city, the capital of the Federal Democratic Republic of Ethiopia from March to April 2018. The city has10 sub-cities and 116 *Woredas* with the total population estimated to be 3,370,778 in 2016 [19]. There are 72 public secondary schools and 103 private schools with 1315 female teachers working in public secondary schools (9–12) [20].

### Study population

All-female teachers in the selected public secondary schools in Addis Ababa were eligible for the study. Among those female teachers who had undergone mastectomy procedure and those who had no one upper extremity were excluded from the study.

### Sample size determination

The sample of 589 female high school teachers was calculated using the formula for a single population proportion considering a 95% confidence interval, 3% margin of error and, the proportion (p) of breast self-examination practice of 12% from a previous study conducted in southern Ethiopia [10] and a design effect of 2. Since total female high school teachers were less than 10,000, a correction formula was used and a10% non-response rate also considered.

### Sampling techniques

A multi-stage sampling method was applied for the selection of the study participants. There are ten sub-cities in Addis Ababa from which Akaki Kality, Yeka, Kolfe and Arada sub-city were selected using the lottery method. All government secondary schools in each selected sub-city were identified and listed. The number of female teachers in the four selected schools was known and the final sample determined by proportionally allocating the female teachers according to their size. Finally, the female teachers were selected from each school by applying simple random sampling technique.

### Measurements

A structured self-administered questionnaire was used to collect information. The questionnaire was adapted from previously published studies and the revised champion’s Health Belief Model (RCHBM) prepared in 1993 and had the following parts; the first part includes socio-demographic background such as age, educational status, marital status, religion, monthly income, teaching experience, personal history of breast problems, and family history of breast cancer; the second part contained questions relateing to the practice of BSE; the third part had questions about knowledge on breast cancer and BSE; the fourth part contained questions on sources of information and the fifth part included questions on the six constructs of the HBM including perceived susceptibility and severity, perceived benefits and barriers, perceived self-efficacy and cues to action questions. The questionnaire was first prepared in the English language then translated to Amharic by a translator. The teachers were given two to three days to complete the questionnaires.

Each question was scored using a 5-point Likert scale ranging from strongly disagree (score 1) to strongly agree (score 5) except for cues to action. Perceived susceptibility contain 3 items ranging from 3 to15, perceived severity-8 items scored from 8 to 40, perceived benefit-6 items scored from 6 to 30, perceived barrier-11 items scored from 11 to 55, self-efficacy-10 items scored from 10 to 50 and cues to action-4 items with ‘yes or no’. We used the sum score of perceived susceptibility plus perceived severity to measure perceived threat and the sum score of perceived benefit minus perceived barrier for perceived net benefit. The knowledge part contained 12 items with ‘yes or no’ answers with 1 score for correct responses and zero score if incorrect.

The questionnaire was pre-tested on 30 female teachers similar to the study population but not included in the actual study. One day of training was given to data collectors by the principal investigator on the objective of the study, contents of the questionnaire and data collection techniques.

### Operational definition

*Breast self-examination practice:* Was assessed as ever practiced BSE and participants who responded as ‘yes’ were considered as practicing BSE.

*Knowledge:* The responses of knowledge items were summed up and mean value was computed from total sum score and was computed for further analysis. It was treated as a continuous variable and higher scores indicated having high knowledge towards practicing BSE.

### Data management and analysis

After data were checked for its completeness and coding the questionnaires, data were entered into Epi-data software version 4.2.0.0 and exported to Statistical Package for Social Sciences (SPSS) version 21 for data analysis. Data were cleaned and negatively worded items were reversely coded before running analysis.

The internal consistency of the subscales of HBM questions was assessed using Cronbach’s alpha reliability coefficient and the result was 0.628 for perceived susceptibility, 0.708 for perceived severity, 0.825 for perceived benefit, 0.892 for perceived barrier and 0.893 for perceived self-efficacy. The assumption of no multi-collinearity between each of the constructs of the Health Belief Model including knowledge was checked and there was no multi-collinearity among the independent variables (VIF < 10).

Descriptive statistics were used to present the results with frequency distribution, proportion, measures of central tendency; perceptions of participants measured based on HBM constructs and were treated as continuous variables, mean and standard deviation were generated for each of HBM constructs. For all constructs of HBM, the responses were summed up and a total score was computed with possible values ranging from minimum to maximum value. The high scores indicated having higher perception towards performing breast self-examination except for barriers in which a higher score indicated a higher barrier to perform BSE. The relationship between each independent variable and the outcome variable of interest (BSE practice) was assessed with binary logistic regression. Those variables found to be associated with the dependent variable in the bivariate analysis (*P* value < 0.25) were entered into multivariable binary logistic regression.

Multivariable binary logistic regression was used to identify the significant predictors of BSE practice after handling possible confounders. The findings were considered at a significant level *p* < 0.05 with a confidence interval (CI) 95%. The Hosmer Lemeshow Goodness of Fit tests were used to check whether the model adequately fits the data in this study. The model adequately fits the data with insignificant value of *p* = 0.31.

## Result

### Socio-demographic characteristics of the study participants

Five hundred and sixty-six female teachers returned their questionnaires, giving a response rate of 96.09%. The age of the study participants ranged from 22 to 59 years with a mean age of 34.19 ± (SD 8.07). The majority of the participants 244 (43.1) were in the age group of 30–39. Over two-thirds of the participants 373(65.9%) were married and 394 (69.6%) were orthodox Christian religion followers. The majority of the respondents, 485 (85.7%) had a first university degree while 354 (62.5%) had less than ten years of experience in teaching. Most of the study participants 533 (94.2%) had no previous history of breast problems (Table 1).

**Table 1:** Socio-demographic characteristics of female secondary school teachers in Addis Ababa, Ethiopia, 2018 (n = 566)

Variables	Frequency	Percentage (%)
*Age (years)*		
20–29	209	36.9
30–39	244	43.1
40–49	80	14.1
> 50	33	5.8
*Education status*		
Diploma	32	5.7
Degree	485	85.7
Second degree	49	8.7
*Marital status*		
Single	153	27
Married	373	65.9
Divorced	25	4.4
Widowed	15	2.7
*Religion*		
Orthodox	394	69.6
Muslim	53	9.4
Protestant	103	18.2
Catholic	13	2.3
Other*	3	0.5
*Monthly income in ETB*		
1st Quartile	143	25.3
2nd Quartile	165	29.2
3rd Quartile	119	21
4th Quartile	139	24.6
*Teaching experience*		
< 10	354	62.5
≥ 10	212	37.5
*History of previous breast disease*		
Yes	33	5.8
No	533	94.2
*Family history of breast cancer*		
Yes	47	8.3
No	519	91.7

*Others Adventist, have no religion, ETB Ethiopian birr

### Source of information and knowledge about breast cancer and breast self-examination

Almost all of the study participants 561 (99.1%) were aware of breast cancer. The majority of the respondents 426 (75.9%) reported they had heard of the breast cancer screening method, breast self-examination (BSE), while 135 (24.1%) female teachers had never heard of it. The mean knowledge score of the study participants was 9.13 (SD ± 4.59).

**Fig. 1:**
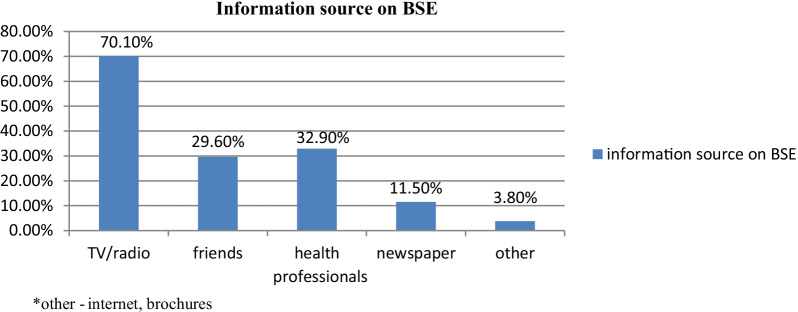
Source of information about breast self-examination (BSE) among female secondary school teachers in Addis Ababa, Ethiopia, 2018 (n = 566)

The most identified sources of information for breast cancer was television and radio (48.3%) followed by friends (22.4%) while sources of information about breast cancer screening measures, breast self-examination (BSE), television, and radio (70.1%) were the main media mentioned followed by friends (29.6%) (Fig. 1).

### Breast self-examination (BSE) practice

Less than half the study participants, 43.6% (95% CI 39.5–47.8%) reported to have examined themselves. Out of those who performed BSE only 25.1% (95% CI 19.8–31%) reported to practice ona monthly interval as recommended (Table 2).

**Table 2:** Practice of breast self-examination among female secondary school teachers Addis Ababa, Ethiopia, 2018 (n = 566)

Variable	Category	Frequency	Percentage (%)
Perform BSE	Yes	247	43.6
	No	319	56.4
Frequency of BSE	Weekly	42	17
	Monthly	62	25
	Every three month	42	17
	Every six month	50	20.2
	Every year	16	6.5
	As I remember	35	14.2
Reason to practice BSE (n = 247)	I have previous self-history of breast problem	8	3.2
	I have family history of breast cancer	11	4.5
	Health professionals recommendation	116	47
	I fear developing breast cancer	112	45.3

### Perception towards BSE

The perception of female teachers on BSE was measured using the constructs of The Health Belief Model and all were analyzed as continuous variables with a mean score of 7.37 (SD ± 2.5) for perceived susceptibility, a mean score of 26.38 (SD ± 5.56) for perceived severity, a mean score 22.87 (SD ± 4.65) for perceived benefits, a mean score of 23.3 (SD ± 8.06) for perceived barrier,and a mean score of 31.37 (SD ± 7.74) for perceived self-efficacy (Table 3).

**Table 3:** Perception of female teachers in Addis Ababa, Ethiopia, 2018 (n = 566)

S. no.	Constructs	Scale range	Mean	SD
1	Perceived susceptibility^a^	3–15	7.37	2.5
2	Perceived severity^a^	8–40	26.38	5.56
3	Perceived benefit^a^	6–30	22.87	4.65
4	Perceived barrier^a^	11–55	23.3	8.06
5	Perceived self-efficacy^a^	10–50	31.37	7.74

*SD* standard deviation

^a^Continuous variable

### Predictors of breast self-examination practice

Bivariate binary logistic regression analysis revealed that marital status, personal history of breast disease, teaching experience, income, age and knowledge were candidates for multiple binary logistic regression analysis at p ≤ 0.25.

Previous history of benign breast disease, experience of teaching, and knowledge of the participants were significantly associated with BSE performance in multivariable binary logistic regression analysis (*p* < 0.05). Female teachers with a previous history of breast problems were 3.27-times more likely to perform BSE than those with no history of any breast disease (95% CI 1.13, 9.45).

It was found that those having ten and more years of teaching experience were 2.46-times more likely to ever perform BSE compared with those who worked less than ten years in the teaching profession (95% CI 1.33, 4.56).

Keeping other variables constant, knowledge was a significant variable in explaining the practice of BSE. A unit increase in the total score of knowledge increased the odds of practicing breast self-examination by 1.07% (95% CI 1.01, 1.12).

Perceived self-efficacy was one of the constructs of the Health Belief Model significantly associated with the practice of BSE. A unit increase in the total score of perceived self-efficacy towards the practice of breast self-examination increases the odds of BSE practice by 7.1% (95% CI 1.01, 1.12) (Table 4).

**Table 4:** Predictors of breast self-examination practice among female secondary school teachers in Addis Ababa, Ethiopia, 2018 (n = 566)

Variables	Practice of BSE		COR (95% CI)	AOR (95% CI)	*P* value
	Yes	No			
*Age*					
20–29	73	136	1	1	
30–39	115	129	1.66 (1.13–2.42)	1.15 (0.66–2.02)	0.606
40–49	39	41	1.77 (1.05–2.98)	0.59 (0.25–1.43)	0.249
> 50	20	13	2.86 (1.34–6.09)	0.84 (0.26–2.68)	0.775
*Marital status*					
Single	55	98	1	1	
Married	174	199	1.55 (1.05–2.29)	1.41 (0.85–2.35)	0.182
Divorced	10	15	1.18 (0.50–2.82)	0.69 (0.22–2.18)	0.535
Widowed	8	7	2.03 (0.70–5.91)	1.48 (0.37–5.88)	0.576
*Personal history of breast problem*					
Yes	22	11	2.73 (1.30–5.76)	3.27 (1.13–9.45)	0.029*
No	225	308	1	1	
*Teaching experience*					
< 10	129	225	1	1	0.004*
≥ 10	118	94	2.19 (1.54–3.09)	2.46 (1.33–4.56)	
*Monthly income*					
1st quadrant	59	84	1	1	
2nd quadrant	55	110	0.71 (0.44–1.13)	0.56 (0.3–1.05)	0.071
3rd quadrant	51	68	1.06 (0.65–1.74)	0.59 (0.28–1.26)	0.179
4th quadrant	82	57	2.04 (1.27–3.29)	0.69 (0.28–1.65)	0.405
Knowledge^a^			1.17 (1.13–1.23)	1.07 (1.01–1.12)	0.009*
Perceived net benefit^a^			1.03 (1.01–1.05)	1.01 (0.98–1.02)	0.588
Perceived self-efficacy^a^			1.09 (1.06–1.12)	1.06 (1.02–1.09)	< 0.001*

*AOR* adjusted odds ratio, *COR* crude odds ratio

1 = Reference category, *indicates statistical significance at *p* < 0.05, ^a^Continuous variable

## Discussion

Less than half the female teachers, 247 (43.6%) reported ever performing BSE, yet 25.1% performed it monthly. Similar findings were observed in previous studies among female teachers in Iran (43%) and Turkey (44%) respectively [21, 22]. The magnitude of BSE performance of this study was higher than the previous study in Kaffa zone, in the southern part of Ethiopia, which was 12% [10], a possible explanati on could be d ifferences in the educational level of the participants, access to information and possible increased breast cancer awareness campaigns in this largely urban sett ing.

In this study, it was found that a history of breast problems was a significant predictor of BSE practice. Study participants who reported having a history of benign breast problems were more likely to practice BSE compared with those with no history of breast problems. This might be due to the experience of such disease making women more aware and ready to get health information regarding breast cancer compared with women who ‘ve never experienced any breast-related health issues [23]. So more likely to perform BSE to detect unusual symptoms. This finding was consistent with a study conducted in Mekelle, northern Ethiopia which also found that a history of previous personal breast problems was significantly associated with performing BSE [14]. Contrary to the present study, a study conducted among female healthcare workers in Turkey showed a previous history of breast problems was not associated with BSE performance [24]. The possible explanation may be those health professionals could be well aware of the benign breast disease and may perceive themselves as not at risk of developing breast cancer and may not, therefore, practice BSE.

The practice of BSE was significantly associated with the teaching experience of the respondents. Those with ten and above years of teaching experience were twice as likely to perform BSE than those with less than ten years. The possible explanation is that female school teachers with more than ten years of work experience are more likely to receive information about BSE due to increased exposure to school-based health education activities compared with those with less than ten years of teaching experience. Inconsistent with our finding, a study carried out in Malaysia and Nigeria found that the length of time spent teaching was not shown to influence BSE performance [18, 24]. This variation could be due to the characteristics of the population being studied. However, the present finding could imply the importance of involving female teachers with great teaching experience in health promotion activities as role models for other young female teachers.

The current study showed that a unit increase in the total score of breast cancer and BSE knowledge increased the odds of practicing BSE by 7%. This may be explained by knowledge of breast cancer and BSE being considered an important precursor to women’s adherence to practicing BSE [10]. It is shown that women who have higher knowledge scores regarding breast cancer and BSE are more likely to practice BSE than women with lower knowledge scores [9]. This signals that in order to increase women’s adherence to BSE performance, more efforts are needed to improve their knowledge about this deadly disease and ways of prevention. This is consistent with other studies [9, 20, 22, 25] conducted among female school teachers.

The other variable significantly associated with the practice of BSE was perceived self-efficacy; a unit increase in the total score of perceived self-efficacy towards breast self-examination increased the odds of practicing BSE by 6.3%. The perceived self-efficacy of women who practiced BSE was higher than those who did not. An explanation could be the assumption of the Health Belief Model, which states that if women have the confidence or greater perception of self-efficacy in their ability to perform BSE; they are more likely to perform BSE than women with lower perceived self-efficacy [26]. A similar finding was also observed in other studies [26–29]. In contrast to our finding, a study conducted in Kaffa zone, Ethiopia reported that perceived self-efficacy and BSE performance were not positively related [10]. This inconsistency might be due to differences in levels of education, and access to information of the study participants.

The current study could not find the association between the perceived threat of breast cancer and the perceived net benefit of practicing BSE with BSE performance which was associated in other studies [10]. Cues to action is another construct of the Health Belief Model that was not a significant predictor of BSE performance. Cues to action may not necessarily influence their screening behavior; there could be other factors such as religious beliefs, lack of skill, or other factors that may influence their behavior. A similar finding was also reported in another study conducted in the southern part of Ethiopia [10].

This study had limitations. The data collection was based on a self-administered questionnaire, teachers were given a few days to complete the questionnaire, making it difficult to control information exchange among the participants. Participants provided self-reported responses regarding their screening behaviors, and no objective measures were used to verify the responses.

## Conclusions

This study aimed to assess the predictors of breast self-examination (BSE) performance among female secondary school teachers based on the Health Belief Model and discovered that the practice of breast self-examination among the participants was low. The perceived self-efficacy, knowledge regarding breast cancer and BSE, previous history of a breast problem, and teaching experience were found to be the predictors of BSE performance.

Educational programs emphasizing improving knowledge regarding breast cancer and BSE should be widely organized to improve the practice of this screening behavior. Training and social and behavioral change communication materials should be prepared and provided aiming to enhance the perceived self-efficacy of teachers towards performing BSE. Therefore, by increasing teachers’ confidence in their ability to perform BSE, it can lead them to positively engage in regular BSE practice. Further research is also recommended in privately owned and other schools to obtain more representative findings.

## Data Availability

The dataset used and analyzed for the current study is not publicly available because we are planning to produce other papers it is available from the corresponding author on reasonable request.

## Abbreviations

ACS: American Cancer Society
AOR: Adjusted odds ratio
BSE: Breast Self-Examination
CBE: Clinical breast examination
CI: Confidence interval
COR: Crude odds ratio
HBM: Health belief model
HCWs: Health care workers
LMICs: Low and middle-income countries
SD: Standard deviation
SPSS: Statistical Package for Social Sciences
RCHBM: Revised Champion's Health Belief Model
ROCs: Reproductive Organ Cancers
